# Supplementary material for: Multireference Protonation Energetics of a Dimeric Model of Nitrogenase Iron–Sulfur Clusters
Source: J Phys Chem A. 2023 Nov 15;127(47):9974–84. doi: 10.1021/acs.jpca.3c06142 (PMC10694817; doi:10.1021/acs.jpca.3c06142)

Supporting Information: Multireference Protonation Energetics of a Dimeric Model of Nitrogenase Iron–Sulfur Clusters

Huanchen Zhai,[1, a)] Seunghoon Lee,[1] Zhi-Hao Cui,[1] Lili Cao,[2] Ulf Ryde,[2, b)] and Garnet Kin-Lic Chan[1, c)]
[1)] *Division of Chemistry and Chemical Engineering, California Institute of Technology, Pasadena, CA 91125, USA*
[2)] *Department of Theoretical Chemistry, Lund University, P. O. Box 124, SE-221 00 Lund, Sweden*

(Dated: 31 October 2023)

I. CLUSTER GEOMETRIES

Cluster geometries are given in XYZ coordinates with the unit Angstrom.

A. Optimized with def2-SV(P)/UKS-TPSS/DFTD3

```
27
HC, Energy = -4717.139080700 H
C    -1.0654597   -1.6165964   -3.6938910
H    -1.4383635   -0.5689779   -3.6741341
H    -1.7807115   -2.2443224   -3.1121367
H    -1.0773907   -1.9924332   -4.7494055
S     0.6243829   -1.6537410   -2.9602556
C     0.4156598    1.7876147   -4.0556776
H     1.3488982    2.3835358   -3.9218054
H    -0.1330190    2.2251636   -4.9293434
H     0.7135941    0.7422418   -4.2919146
S    -0.6070437    1.7896179   -2.5222937
C    -0.8232495    2.8573739    1.4681234
H    -0.0877151    2.8066116    0.6407689
H    -0.9439809    3.9246222    1.7897946
H    -1.7975667    2.5226444    1.0498580
S    -0.2735647    1.7807874    2.8579066
C    -1.0643237   -1.5242334    4.0337374
H    -1.0304628   -1.8368555    5.1103107
H    -1.8734238   -2.1231958    3.5494502
H    -1.3541746   -0.4526895    3.9781077
S     0.5588840   -1.7538671    3.1864833
Fe    0.4943827    0.0464709   -1.2052434
Fe    0.5810012   -0.0475200    1.4684925
S     2.2702018    0.5987571    0.0818694
C    -0.9804810   -0.8504359    0.1595883
H    -0.9278775   -1.9603768    0.2106940
H    -1.5301625   -0.5677658   -0.7796813
H    -1.6330329   -0.4914309    0.9945982
```

Listing S1. HC

```
27
HS, Energy = -4717.096074143 H
C    -1.4481492   -1.2918793   -4.0685921
H    -1.5592125   -0.1859823   -4.0893224
H    -2.3309980   -1.7079134   -3.5297987
H    -1.4760151   -1.6885040   -5.1175674
S     0.1243831   -1.7171690   -3.2045707
C     1.0341701    1.7983129   -3.6269368
```

a) Electronic mail: hczhai.ok@gmail.com
b) Electronic mail: ulf.ryde@compchem.lu.se
c) Electronic mail: gkc1000@gmail.com

```
H      1.9627435     1.9656559    -3.0387507
H      0.9926332     2.5651117    -4.4445661
H      1.1042400     0.7824705    -4.0723829
S     -0.4446417     1.8649325    -2.5294114
C     -1.1427404     2.6271747     1.3777265
H     -0.7051544     2.6282955     0.3581567
H     -1.2853067     3.6896251     1.7066593
H     -2.1508112     2.1651199     1.2944240
S     -0.0778785     1.6928495     2.5587062
C     -0.7929520    -1.4324890     4.2467679
H     -0.9625390    -0.3357608     4.1885620
H     -0.5400427    -1.7119510     5.3045419
H     -1.7588854    -1.9373028     4.0010839
S      0.5386887    -1.8989304     3.0569739
Fe    -0.0624496    -0.2245743    -1.3014183
Fe     0.1805793    -0.3587873     1.2612831
S      2.1356417     0.1952062    -0.2457191
C     -1.2915962    -0.9617811     0.0689167
H      1.9086364     1.5088483     0.0713138
H     -2.2983991    -0.4932717     0.2239739
H     -1.4219432    -2.0743068     0.0479475
```

Listing S2. HS

```
27
HFe, Energy = -4717.084045403 H
C     -1.7030912    -0.2687576    -3.8857510
H     -1.4276567     0.6161659    -3.2739484
H     -2.7282115    -0.5817080    -3.5791673
H     -1.7544034     0.0472283    -4.9585791
S     -0.4654014    -1.5971491    -3.6068456
C      1.3427752     1.2911521    -3.6530591
H      2.4027725     1.5361646    -3.9146487
H      0.7051324     2.0932763    -4.0999022
H      1.0611069     0.3183708    -4.1168671
S      1.1257526     1.1531598    -1.8302475
C     -0.5460690     2.6148722     1.2254132
H      0.3817785     2.2964072     1.7410684
H     -0.7581861     3.6865849     1.4682270
H     -0.3516724     2.5166490     0.1383690
S     -1.9524226     1.5437145     1.7437219
C     -1.2593992    -1.2001762     4.2059136
H     -0.1794134    -1.4477025     4.1290192
H     -1.6496014    -1.5773749     5.1869269
H     -1.3493637    -0.0933792     4.1760396
S     -2.1872294    -1.9182902     2.7849438
Fe     0.2293122    -1.0722584    -1.1389980
Fe    -1.0895429    -0.6236298     1.1100321
S      1.1201274    -0.6801252     1.0718109
C     -1.6833719    -1.3940353    -0.5791815
H      1.1279016    -2.5680078    -1.3656985
H     -2.5031122    -0.8682227    -1.1171969
H     -1.7445096    -2.4939284    -0.7433938
```

Listing S3. HFe

```
27
HFe2, Energy = -4717.102540727 H
C     -0.6004690    -1.8541396    -4.3931123
H     -0.9885128    -0.8116508    -4.4441158
H     -1.2233186    -2.5109691    -5.0545639
H      0.4332653    -1.8614472    -4.8208224
S     -0.6420813    -2.4169491    -2.6407698
C      0.4911160     1.7119732    -3.4952934
H      1.1454118     1.7908560    -2.6017265
H      0.4940557     2.6834804    -4.0526033
H      0.9297739     0.9375322    -4.1667404
S     -1.2045031     1.2624527    -2.9490191
C     -0.5170233     2.7735147     1.7890395
H      0.1897341     2.5579946     0.9587402
H     -0.1817476     3.6864329     2.3434608
```

```
H     -1.5092436     2.9968065     1.3360800
S     -0.5859628     1.3069271     2.8986914
C     -0.5055631    -1.6440394     4.3765240
H     -1.3125977    -0.8903412     4.5041769
H      0.4429013    -1.1359999     4.6628636
H     -0.6817372    -2.4716890     5.1124802
S     -0.4582362    -2.2773941     2.6480953
Fe    -0.5822491    -0.4261876    -1.2438221
Fe    -0.4040092    -0.3452652     1.1749566
S      1.3672017     0.2724370    -0.2315191
C     -2.0078868     0.0019752     0.0519971
H     -0.5166457    -1.6358452    -0.0228939
H     -2.3493298     1.0582445    -0.0027923
H     -2.8433420    -0.7077099     0.2366894
```

Listing S4. HFe_2

B. Optimized with def2-TZVP/UKS-TPSS/DFTD3

```
27
HC, Energy =    -5076.012745 H
C      -1.89342031    -2.94949331    -7.12177899
H      -2.54339731    -0.97906391    -7.09286332
H      -3.30953872    -4.10746983    -6.13271285
H      -1.78233191    -3.61259806    -9.09765127
S       1.20164028    -3.14391237    -5.53655396
C       0.70175561     3.31623544    -7.75340011
H       2.42278875     4.48645307    -7.75437981
H      -0.55001429     4.01726270    -9.26916545
H       1.25567282     1.35936496    -8.16726945
S      -0.88385618     3.42838856    -4.65419662
C      -1.59341328     5.38738577     2.69823716
H      -0.16329228     5.34717827     1.20448660
H      -1.86184135     7.35300309     3.34781319
H      -3.37275285     4.72943706     1.86139269
S      -0.60598410     3.33049089     5.32345806
C      -1.97412845    -2.92554715     7.85809788
H      -1.77207663    -3.52650830     9.84758262
H      -3.52264699    -4.03137012     7.01379265
H      -2.50137471    -0.92207831     7.79977086
S       0.99725315    -3.37479862     6.08671301
Fe      1.00043688     0.01396551    -2.23952733
Fe      1.02807517    -0.20085270     2.85930267
S       4.26540766     0.81314534     0.32021427
C      -1.93759727    -1.49377793     0.28393699
H      -1.95616552    -3.56679309     0.32360730
H      -2.89342485    -0.84128190    -1.46884922
H      -3.10644747    -0.76171110     1.85525513
```

Listing S5. HC

```
27
HS, Energy =    -5076.012745 H
C      -2.28190831    -2.62210518    -7.76955353
H      -2.56284663    -0.56787565    -7.85553475
H      -4.03818994    -3.47510426    -7.05136733
H      -1.94432387    -3.35920341    -9.69484777
S       0.40213316    -3.31569812    -5.65738597
C       1.57606301     3.29373354    -7.35844928
H       3.42391523     3.96597767    -6.68727406
H       1.01832504     4.44632529    -9.00859059
H       1.79096721     1.31185662    -7.93261306
S      -0.79847573     3.47813910    -4.81583865
C      -2.08354987     5.15638282     2.93197739
H      -1.21863646     5.12371124     1.05214145
H      -2.16709842     7.13010952     3.60467260
H      -4.02585861     4.45405403     2.73259777
S      -0.27975217     3.16867996     5.15203571
```

```
C      -1.59027439   -3.03107071    8.07979081
H      -1.89832095   -0.98326605    8.17172587
H      -0.97425784   -3.72263174    9.95295889
H      -3.40997359   -3.94032514    7.63279479
S       0.79130768   -3.71820521    5.62317417
Fe     -0.03478993   -0.25266009   -2.35732660
Fe      0.26452015   -0.52658586    2.52302989
S       3.94044001    0.17720368   -0.16367398
C      -2.57249201   -1.25056306    0.19231189
H       3.82334645    2.70489675    0.20264152
H      -4.27428958   -0.05798262    0.40347862
H      -3.11427509   -3.26422004    0.14094785
```

Listing S6. HS

```
27
HFe, Energy =   -5076.012745 H
C      -3.75913157   -2.73243516   -5.62379855
H      -3.29406598   -0.86937361   -6.40698422
H      -5.34465440   -2.50613889   -4.30508511
H      -4.33389115   -4.02169448   -7.16164635
S      -0.94169345   -3.95902372   -4.00566722
C       3.36704638    0.78931871   -6.64033009
H       5.16356944    0.74240615   -5.59643989
H       3.70490535    1.68962586   -8.49608837
H       2.74508794   -1.16929167   -6.92812697
S       0.93491439    2.49051761   -4.84940786
C      -1.68456058    7.18270902    2.40049702
H       0.19224944    6.49260250    1.86699529
H      -1.50677428    9.06394023    3.29003486
H      -2.79670363    7.39397233    0.65959374
S      -3.19217575    4.89925564    4.56992044
C      -2.19692263   -4.80564329    4.43881601
H      -2.57539687   -4.92192958    2.40602069
H      -3.03292045   -6.46935583    5.38725653
H      -0.13958047   -4.84884165    4.68075772
S      -3.48483113   -1.83709967    5.73824166
Fe      0.38826032   -0.59158352   -1.35560261
Fe     -1.70350676    1.07471666    2.92488570
S       2.41535463    0.66757931    2.06352051
C      -3.11193692    0.30561124   -0.38195714
H       2.67456217   -2.54093001   -1.48834688
H      -3.80956887    2.12943289   -1.11041352
H      -4.60333594   -1.13690125   -0.42009557
```

Listing S7. HFe

```
27
HFe2, Energy =   -5076.012745 H
C      -1.10308696   -3.43573011   -8.31015755
H      -1.97612529   -1.55868177   -8.47300817
H      -2.14811060   -4.78815975   -9.50868326
H       0.84450816   -3.32220378   -9.04341854
S      -1.16558991   -4.42709801   -4.97779147
C       1.01349275    2.98702318   -6.87382013
H       2.38288989    3.07635358   -5.32040867
H       1.08693632    4.76165870   -7.96939213
H       1.55847962    1.42276663   -8.12966587
S      -2.15634240    2.50020374   -5.54907517
C      -1.11945035    5.29777671    3.59413326
H       0.39205944    5.04544188    2.19778930
H      -0.71668237    6.96183152    4.78580215
H      -2.88709716    5.63793316    2.56070368
S      -1.34233521    2.43107787    5.53953962
C      -0.81834672   -3.19565308    8.30207207
H      -2.35902855   -1.86830822    8.70843936
H       0.96112552   -2.20397624    8.69784276
H      -0.97571351   -4.81794673    9.60760604
S      -0.94162510   -4.27161064    4.99428938
Fe     -1.11154921   -0.67337271   -2.32791573
Fe     -0.91042029   -0.60040006    2.27934685
```

```
S        2.47213018     0.49721313    -0.26094746
C       -3.82542178     0.21332099     0.05633629
H       -1.08992756    -2.97599580    -0.02897393
H       -4.34227128     2.22216152     0.00023204
H       -5.46245532    -1.03979771     0.28218450
```

Listing S8. HFe_2

II. DMRG ENERGY EXTRAPOLATIONS

The following figures show the DMRG energy extrapolation process for the four protonated structures HC, HS, HFe, and HFe_2 in the (36o, 48e), (55o, 48e), and (63o, 64e) active spaces.

A. The (36o, 48e) active space

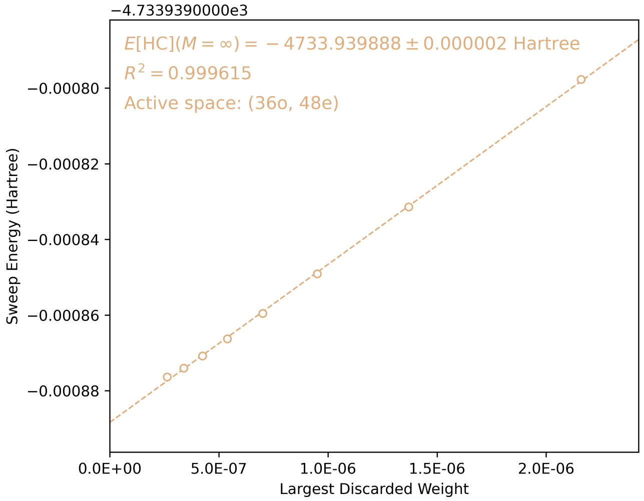

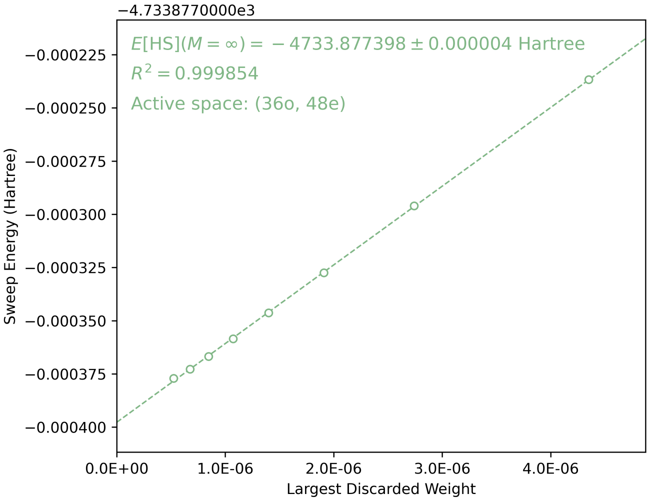

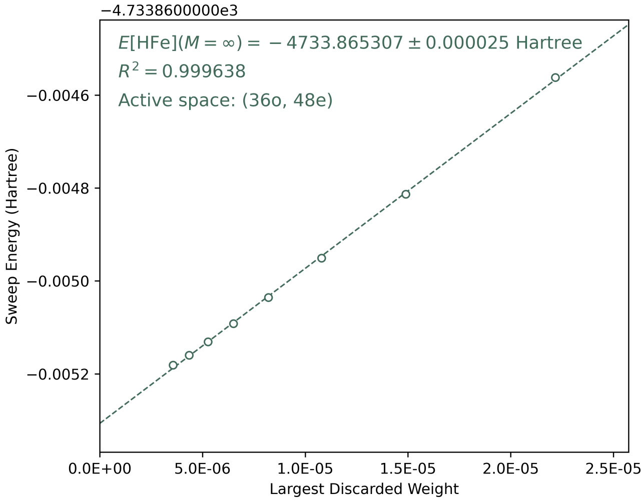

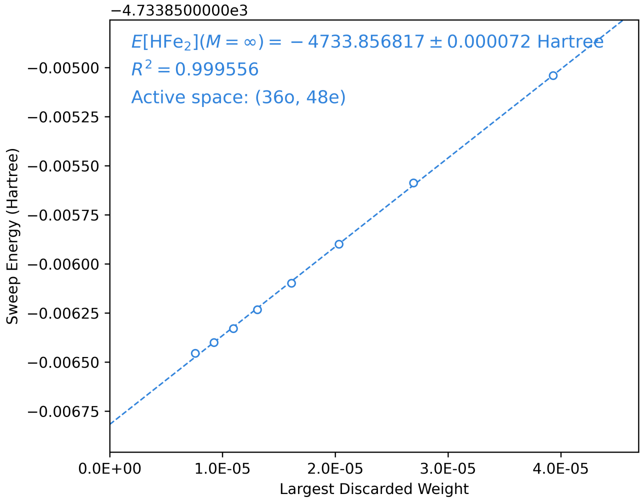

B. The (55o, 48e) active space

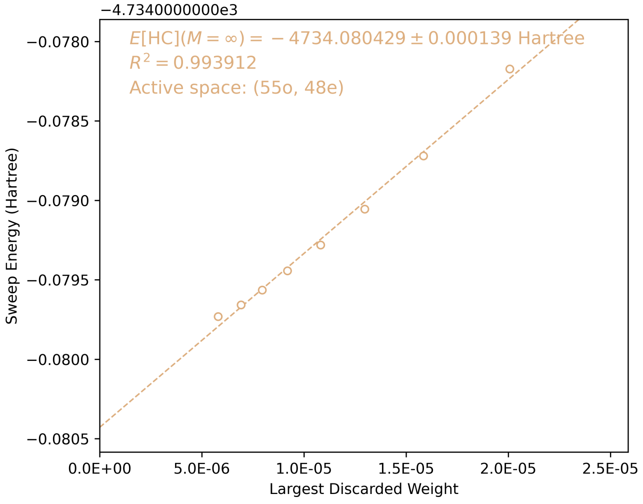

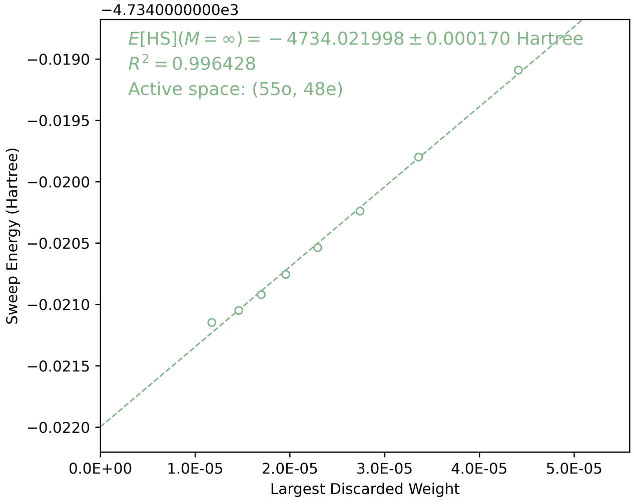

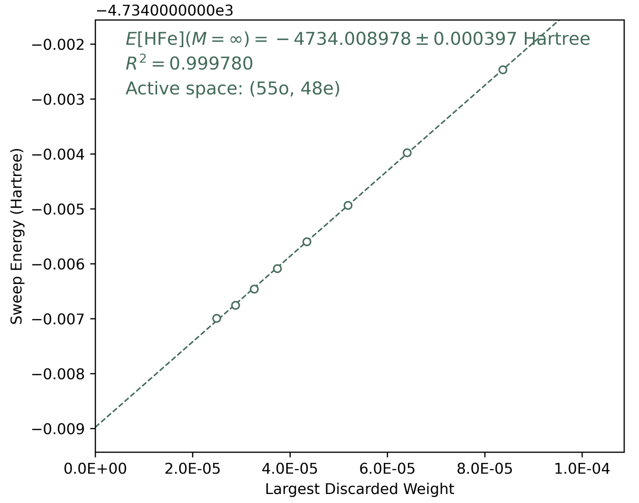

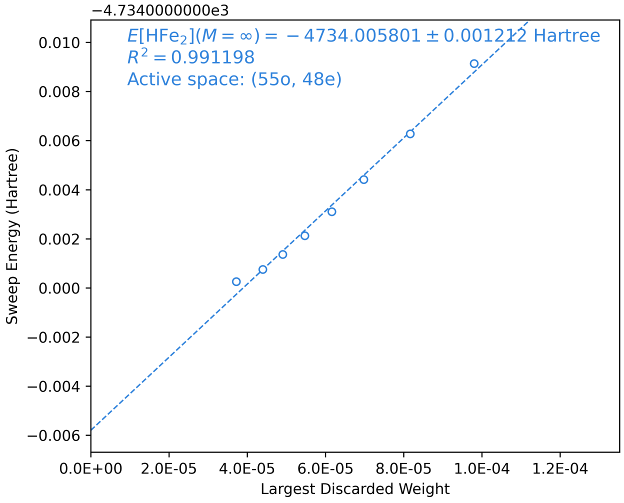

C. The (63o, 64e) active space

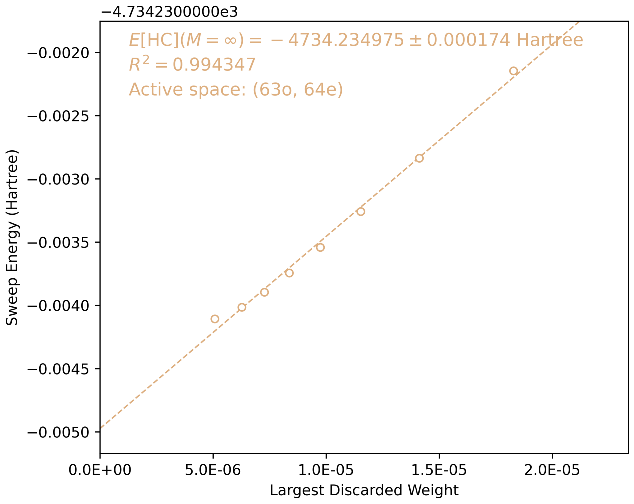

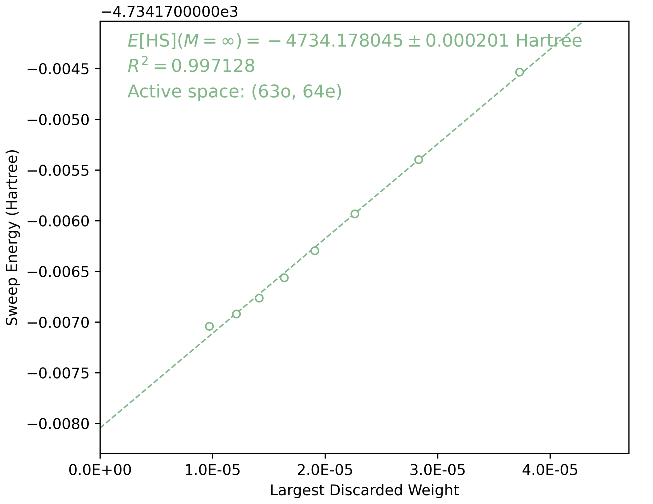

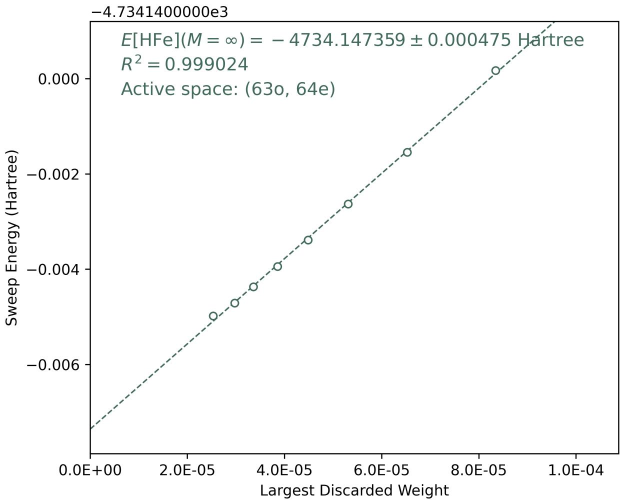

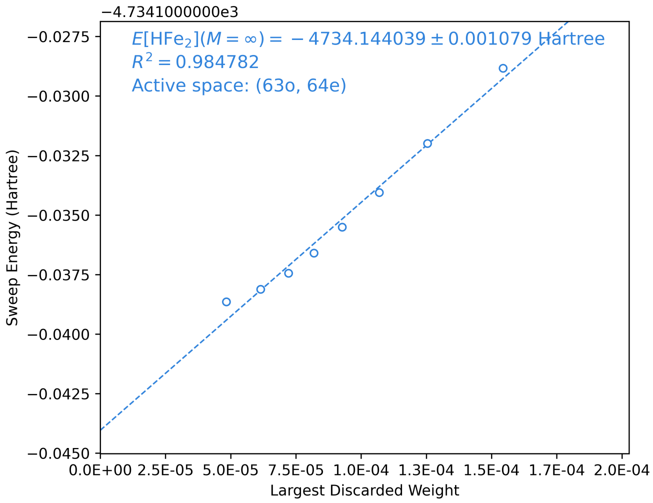

III. CCSD NATURAL ORBITALS

The following figures show the CCSD natural orbitals for constructing the active space. Orbitals are ordered according to the CCSD natural occupation number from highest to lowest, counting from zero. Orbital #86∼#93 are close to singly occupied. The (36o, 48e) active space consists orbitals #66∼#101. The (55o, 48e) active space consists orbitals #66∼#120. The (63o, 64e) active space consists orbitals #58∼#120.

A. HC

[HC- 58] occ = 1.963

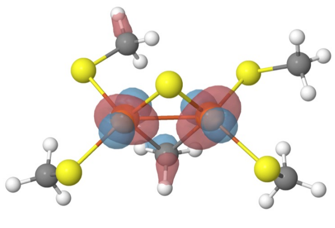

[HC- 59] occ = 1.961

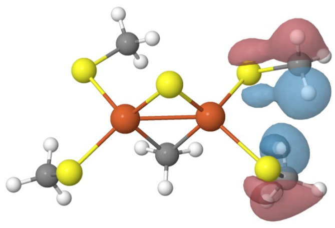

[HC- 60] occ = 1.960

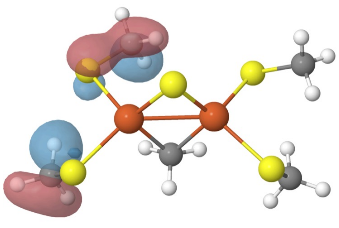

[HC- 61] occ = 1.959

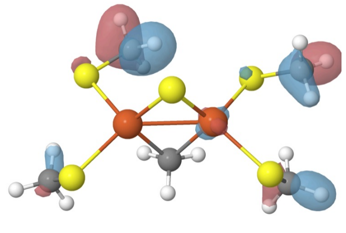

[HC- 62] occ = 1.959

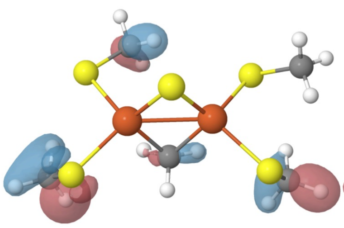

[HC- 63] occ = 1.959

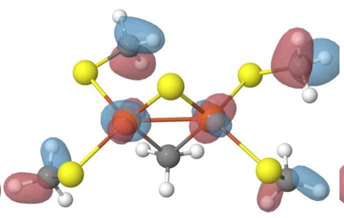

[HC- 64] occ = 1.958

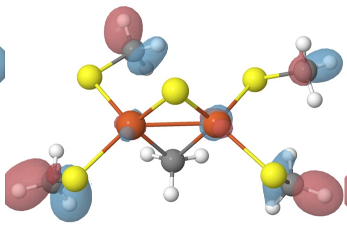

[HC- 65] occ = 1.958

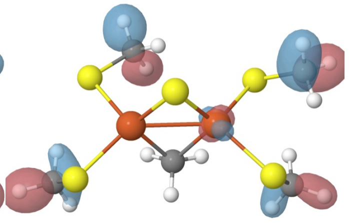

[HC- 66] occ = 1.958

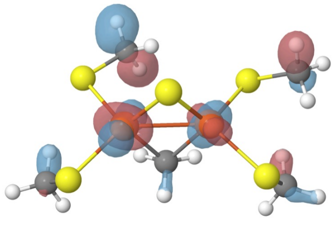

[HC- 67] occ = 1.957

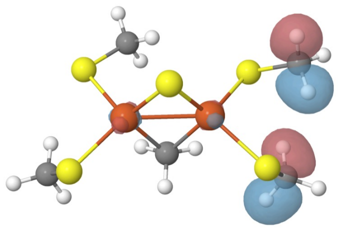

[HC- 68] occ = 1.956

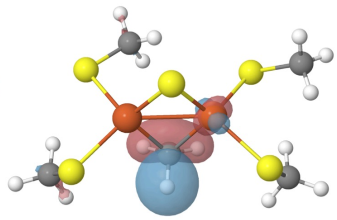

[HC- 69] occ = 1.955

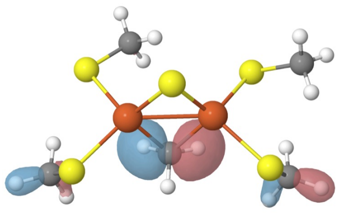

[HC- 70] occ = 1.952

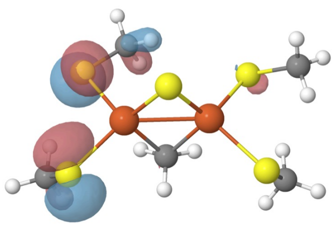

[HC- 71] occ = 1.951

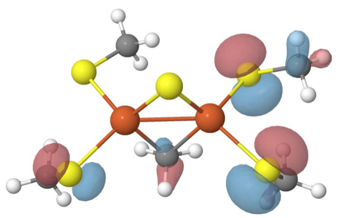

[HC- 72] occ = 1.951

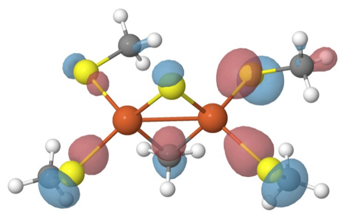

[HC- 73] occ = 1.950

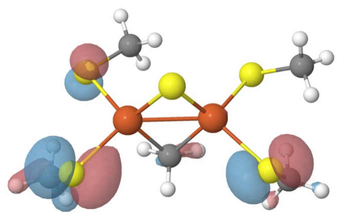

[HC- 74] occ = 1.950

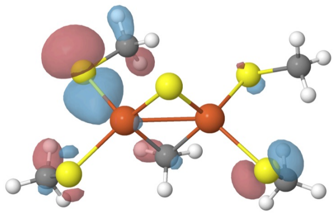

[HC- 75] occ = 1.949

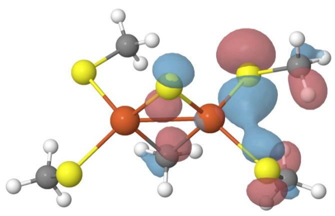

[HC- 76] occ = 1.949

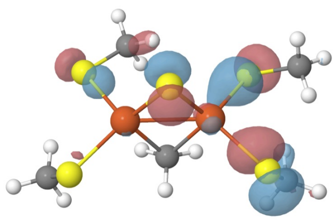

[HC- 77] occ = 1.948

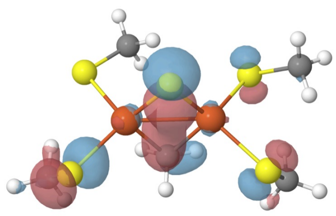

[HC- 78] occ = 1.948

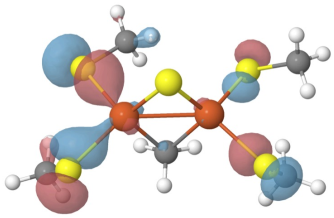

[HC- 79] occ = 1.946

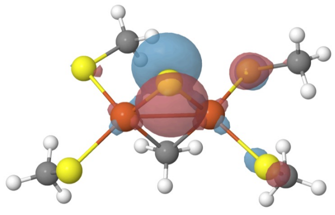

[HC- 80] occ = 1.944

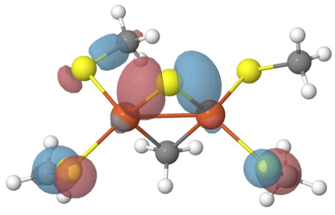

[HC- 81] occ = 1.943

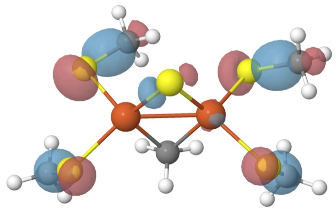

[HC- 82] occ = 1.942

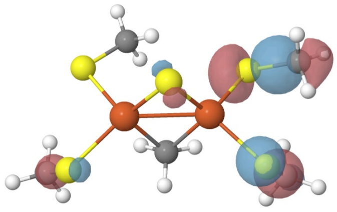

[HC- 83] occ = 1.942

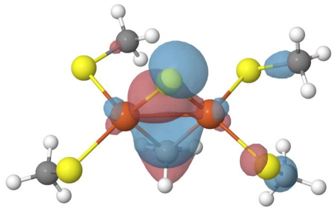

[HC- 84] occ = 1.942

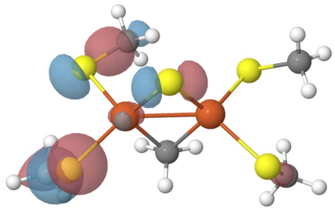

[HC- 85] occ = 1.941

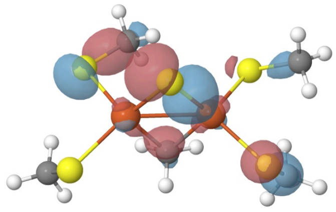

[HC- 86] occ = 1.162

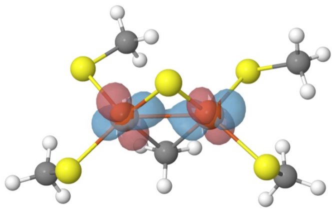

[HC- 87] occ = 1.148

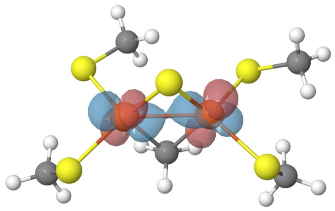

[HC- 88] occ = 1.032

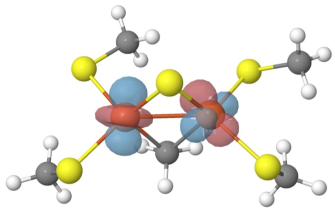

[HC- 89] occ = 1.021

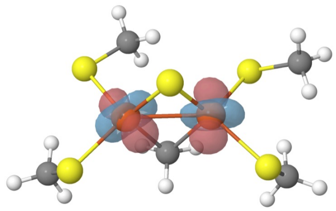

[HC- 90] occ = 0.971

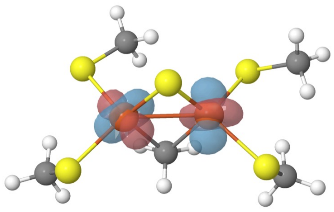

[HC- 91] occ = 0.962

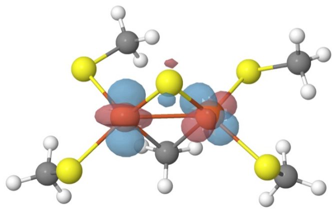

[HC- 92] occ = 0.847

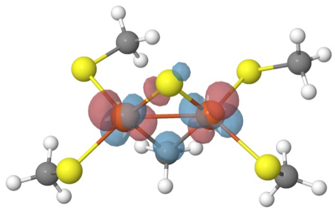

[HC- 93] occ = 0.833

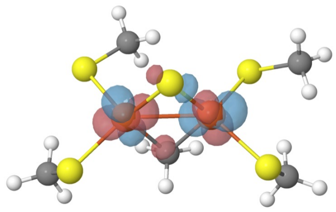

[HC- 94] occc = 0.051

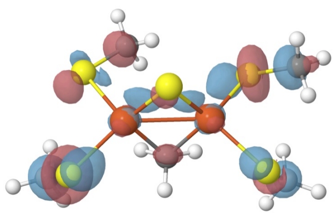

[HC- 95] occ = 0.048

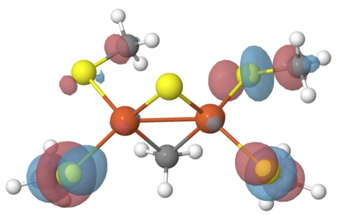

[HC- 96] occ = 0.044

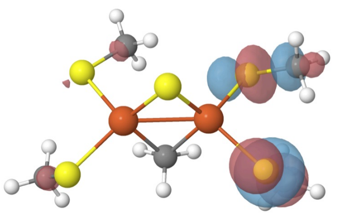

[HC- 97] occ = 0.044

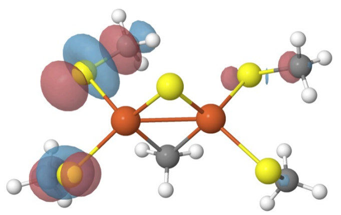

[HC- 98] occ = 0.038

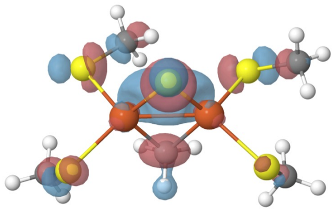

[HC- 99] occ = 0.032

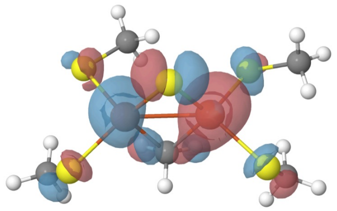

[HC-100] occ = 0.031

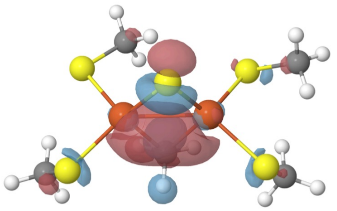

[HC-101] occ = 0.029

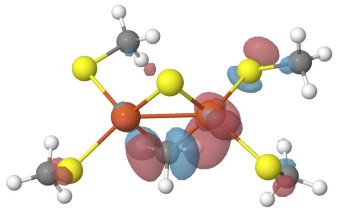

[HC-102] occ = 0.028

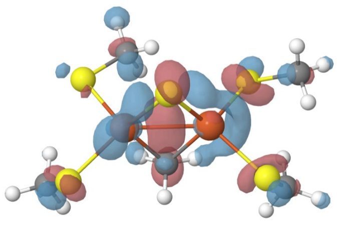

[HC-103] occ = 0.028

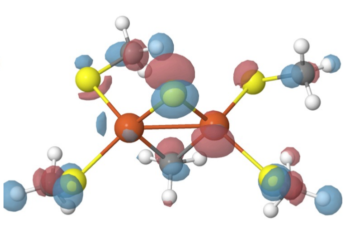

[HC-104] occ = 0.027

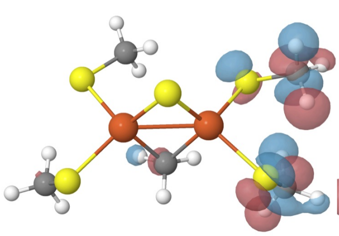

[HC-105] occ = 0.027

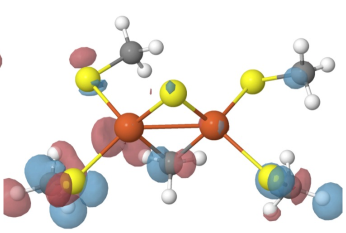

[HC-106] occ = 0.027

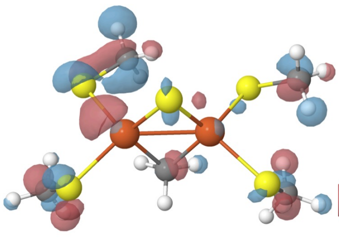

[HC-107] occ = 0.026

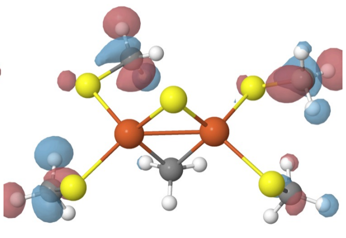

[HC-108] occ = 0.026

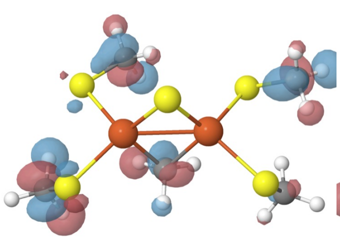

[HC-109] occ = 0.026

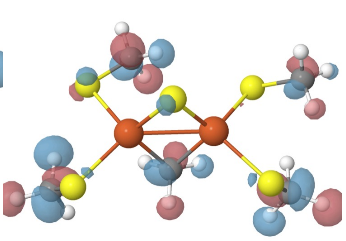

[HC-110] occ = 0.025

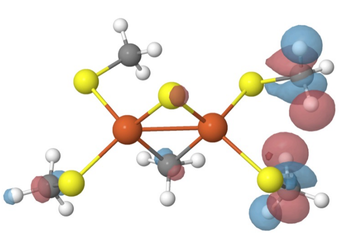

[HC-111] occ = 0.025

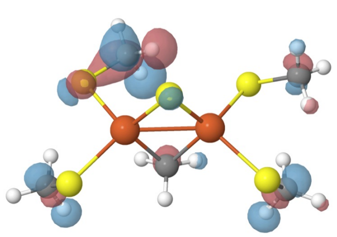

[HC-112] occ = 0.025

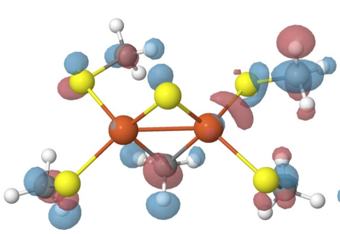

[HC-113] occ = 0.024

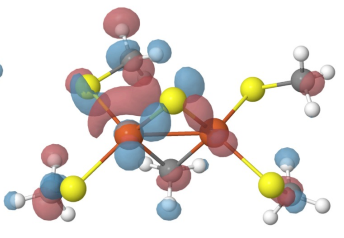

[HC-114] occ = 0.024

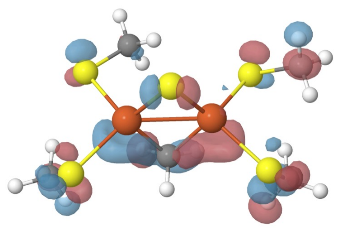

[HC-115] occ = 0.023

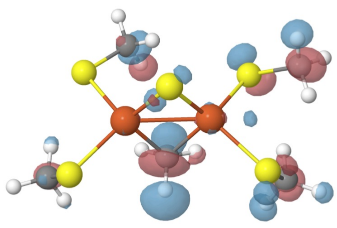

[HC-116] occ = 0.022

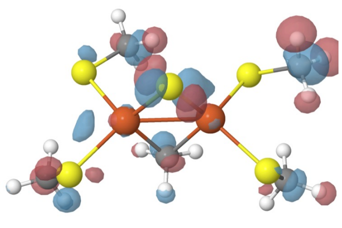

[HC-117] occ = 0.022

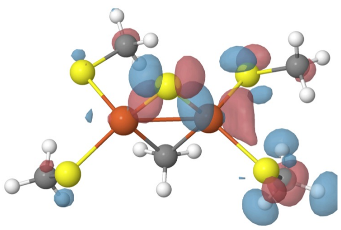

[HC-118] occ = 0.022

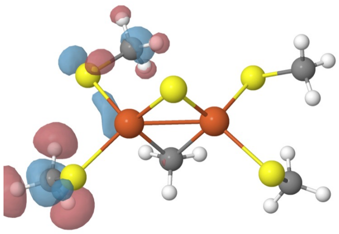

[HC-119] occ = 0.021

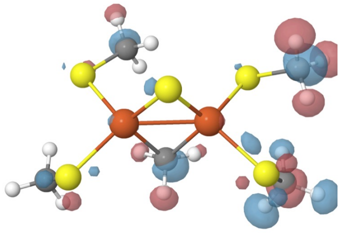

[HC-120] occ = 0.021

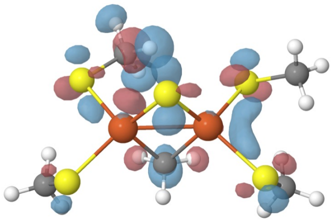

B. HS

[HS- 58] occ = 1.961

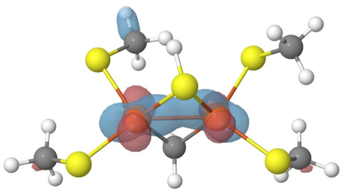

[HS- 59] occ = 1.961

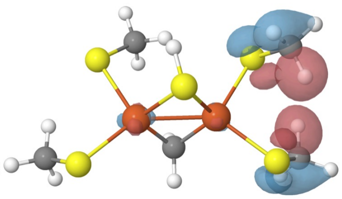

[HS- 60] occ = 1.960

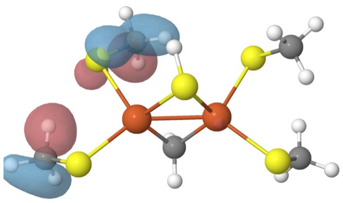

[HS- 61] occ = 1.959

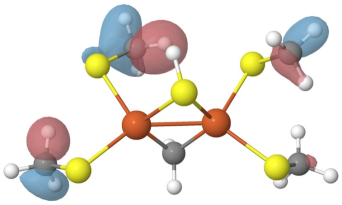

[HS- 62] occ = 1.958

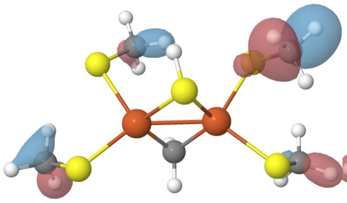

[HS- 63] occ = 1.958

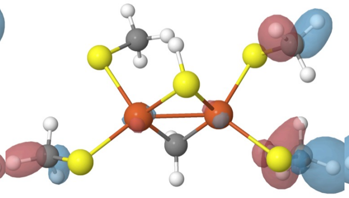

[HS- 64] occ = 1.958

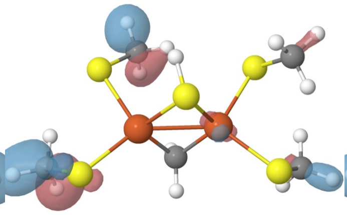

[HS- 65] occ = 1.958

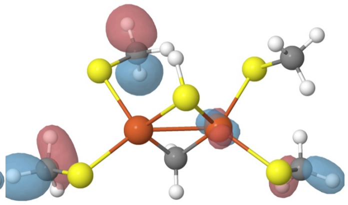

[HS- 66] occ = 1.958

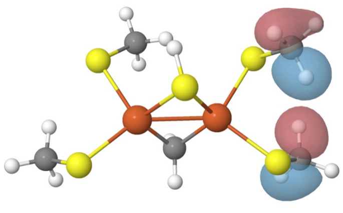

[HS- 67] occ = 1.957

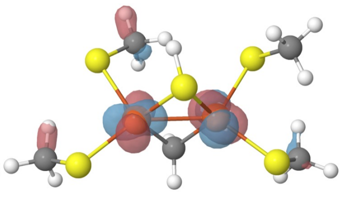

[HS- 68] occ = 1.955

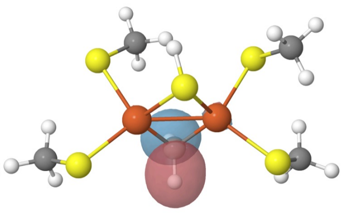

[HS- 69] occ = 1.952

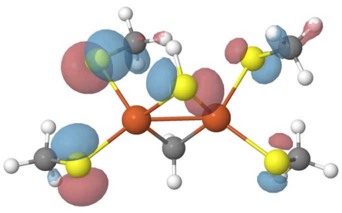

[HS- 70] occ = 1.951

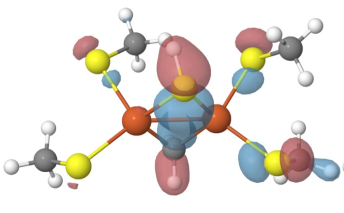

[HS- 71] occ = 1.951

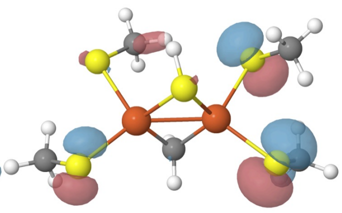

[HS- 72] occ = 1.950

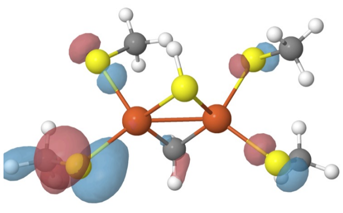

[HS- 73] occ = 1.950

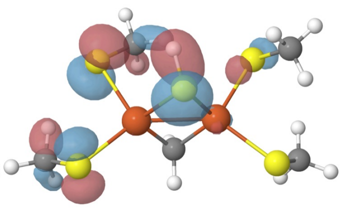

[HS- 74] occ = 1.949

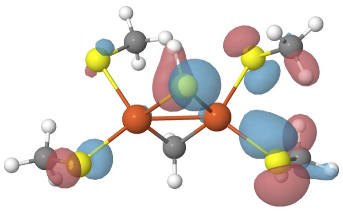

[HS- 75] occ = 1.949

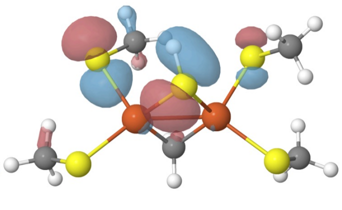

[HS- 76] occ = 1.949

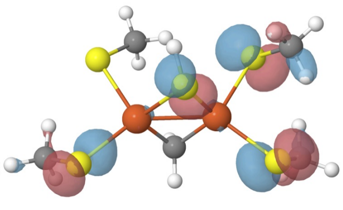

[HS- 77] occ = 1.948

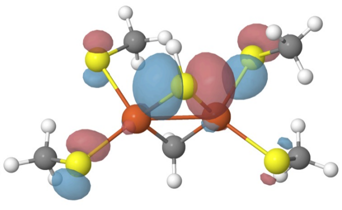

[HS- 78] occ = 1.947

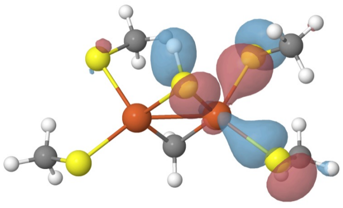

[HS- 79] occ = 1.947

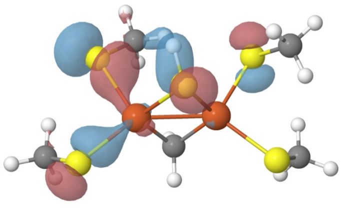

[HS- 80] occ = 1.944

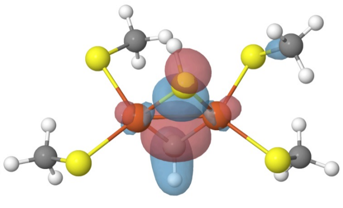

[HS- 81] occ = 1.943

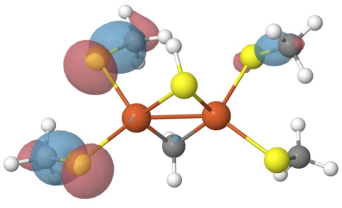

[HS- 82] occ = 1.943

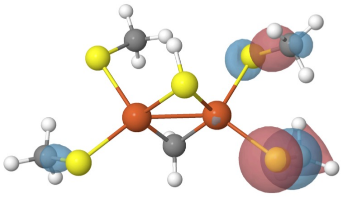

[HS- 83] occ = 1.942

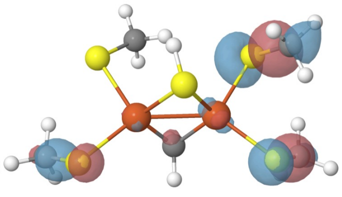

[HS- 84] occ = 1.942

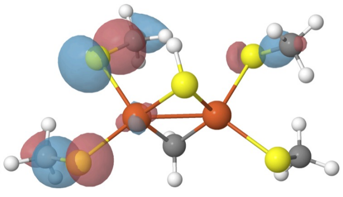

[HS- 85] occ = 1.924

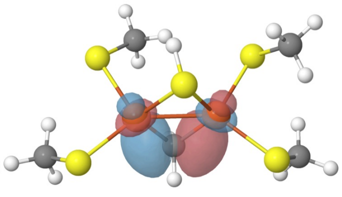

[HS- 86] occ = 1.317

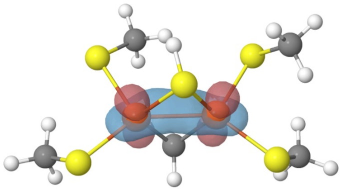

[HS- 87] occ = 1.103

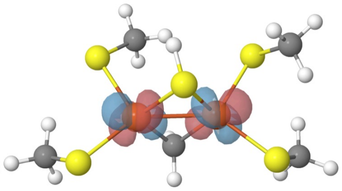

[HS- 88] occ = 1.089

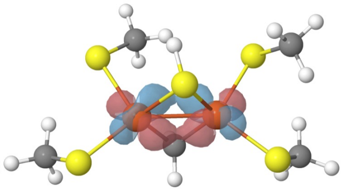

[HS- 89] occ = 0.998

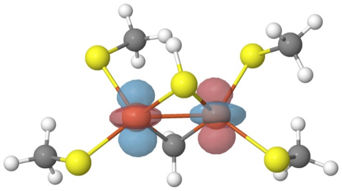

[HS- 90] occ = 0.991

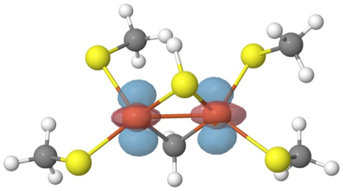

[HS- 91] occ = 0.900

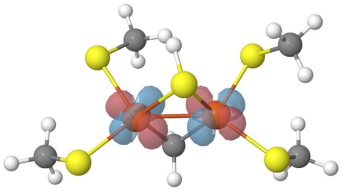

[HS- 92] occ = 0.891

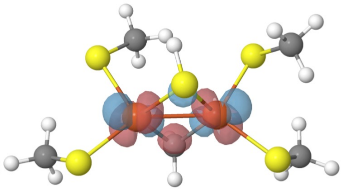

[HS- 93] occ = 0.685

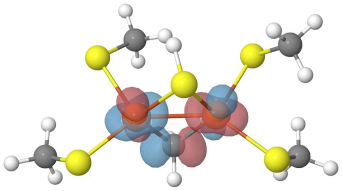

[HS- 94] occ = 0.052

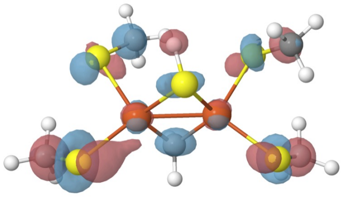

[HS- 95] occ = 0.049

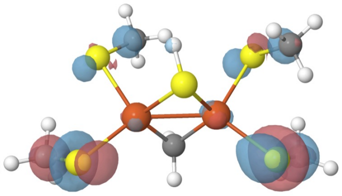

[HS- 96] occ = 0.046

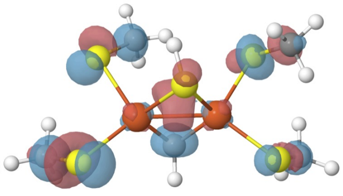

[HS- 97] occ = 0.045

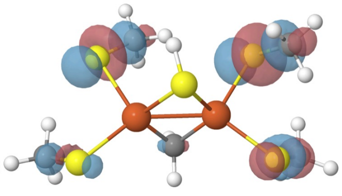

[HS- 98] occ = 0.042

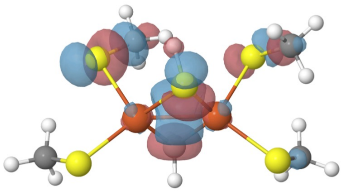

[HS- 99] occ = 0.040

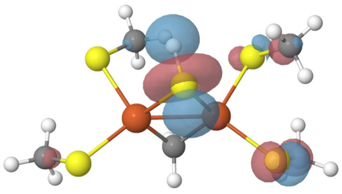

[HS-100] occ = 0.033

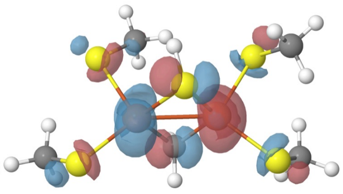

[HS-101] occ = 0.031

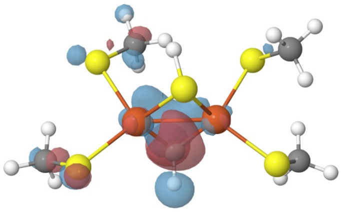

[HS-102] occ = 0.029

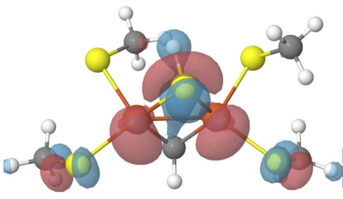

[HS-103] occ = 0.028

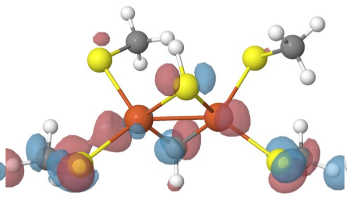

[HS-104] occ = 0.028

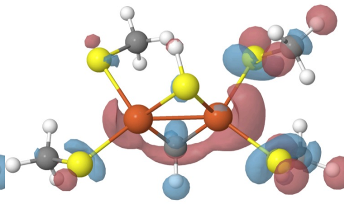

[HS-105] occ = 0.027

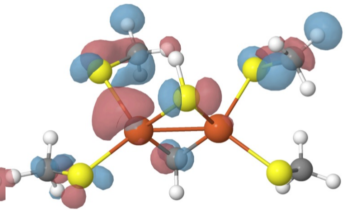

[HS-106] occ = 0.027

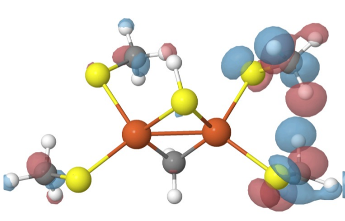

[HS-107] occ = 0.026

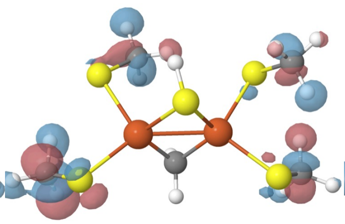

[HS-108] occ = 0.026

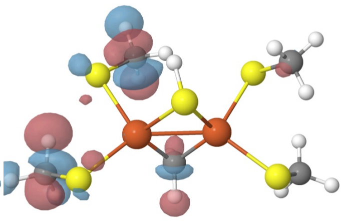

[HS-109] occ = 0.026

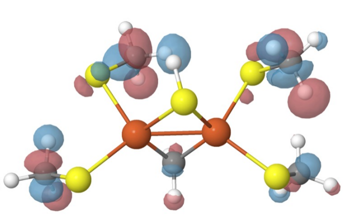

[HS-110] occ = 0.026

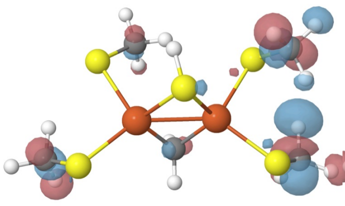

[HS-111] occ = 0.026

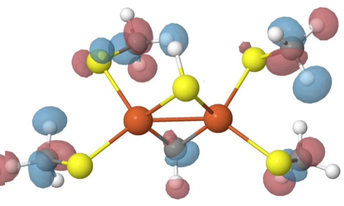

[HS-112] occ = 0.025

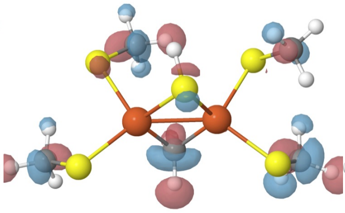

[HS-113] occ = 0.024

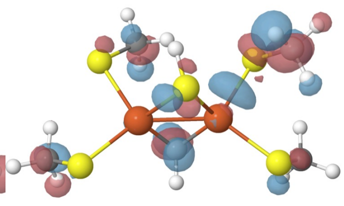

[HS-114] occ = 0.024

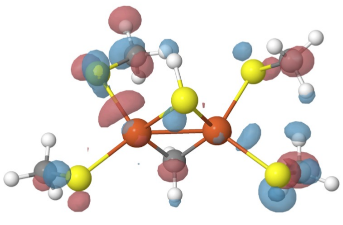

[HS-115] occ = 0.024

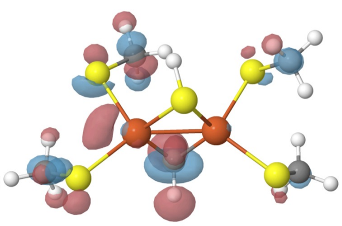

[HS-116] occ = 0.023

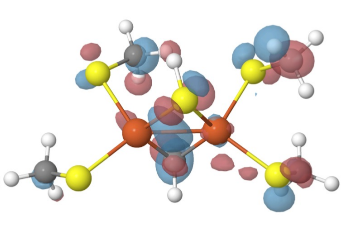

[HS-117] occ = 0.022

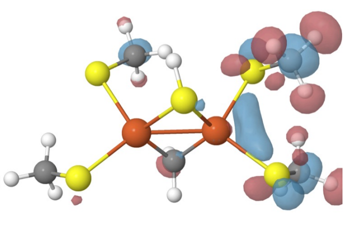

[HS-118] occ = 0.022

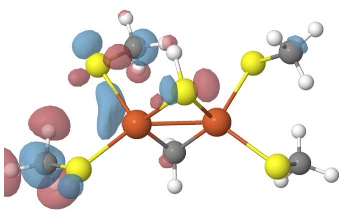

[HS-119] occ = 0.021

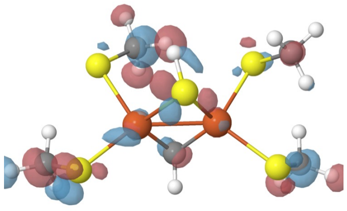

[HS-120] occ = 0.021

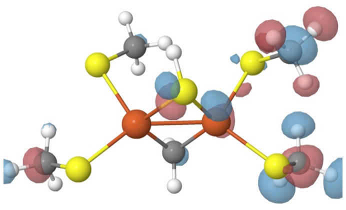

C. HFe

[HFe- 58] occ = 1.961

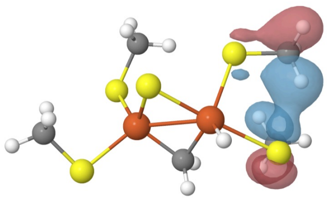

[HFe- 59] occ = 1.960

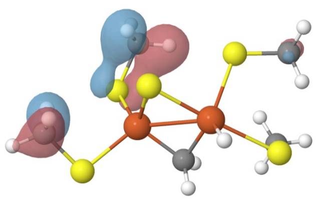

[HFe- 60] occ = 1.959

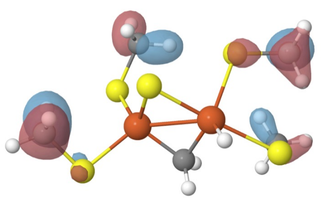

[HFe- 61] occ = 1.959

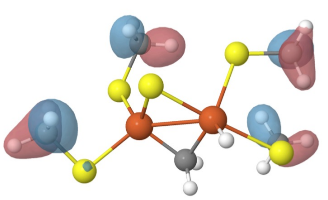

[HFe- 62] occ = 1.959

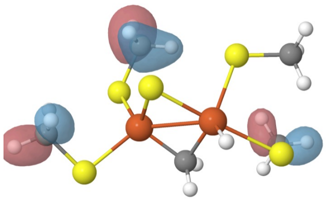

[HFe- 63] occ = 1.958

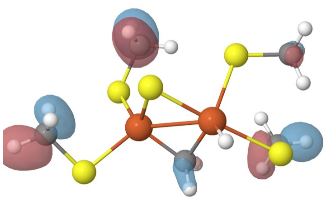

[HFe- 64] occ = 1.958

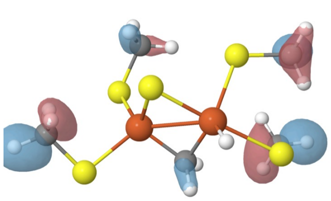

[HFe- 65] occ = 1.957

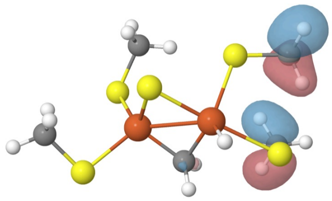

[HFe- 66] occ = 1.956

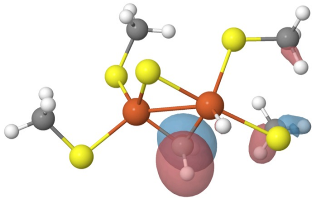

[HFe- 67] occ = 1.952

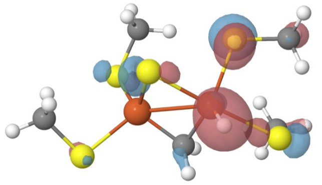

[HFe- 68] occ = 1.951

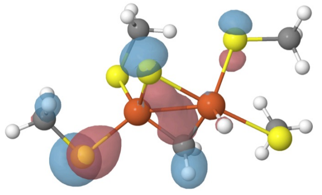

[HFe- 69] occ = 1.951

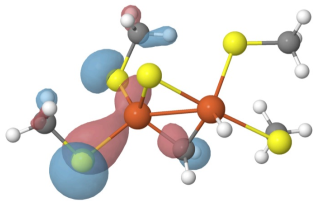

[HFe- 70] occ = 1.950

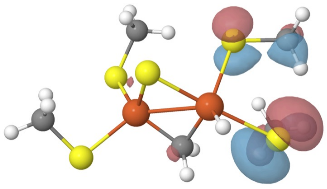

[HFe- 71] occ = 1.950

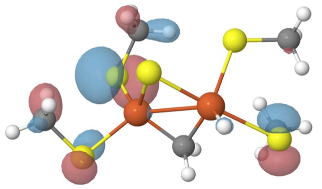

[HFe- 72] occ = 1.949

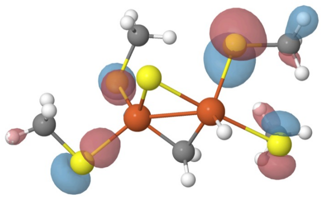

[HFe- 73] occ = 1.948

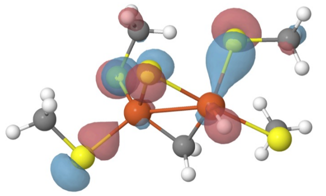

[HFe- 74] occ = 1.947

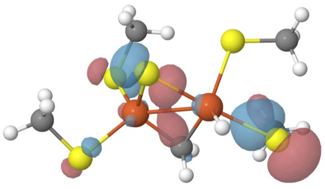

[HFe- 75] occ = 1.946

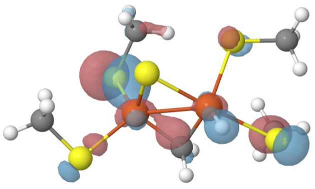

[HFe- 76] occ = 1.945

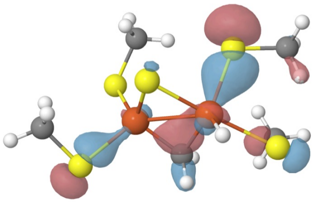

[HFe- 77] occ = 1.944

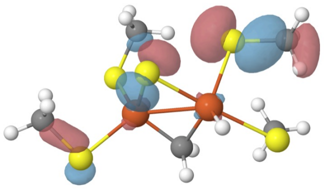

[HFe- 78] occ = 1.943

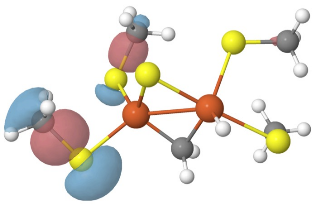

[HFe- 79] occ = 1.943

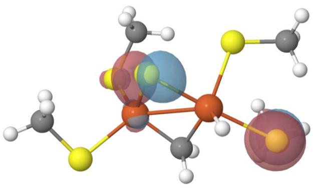

[HFe- 80] occ = 1.942

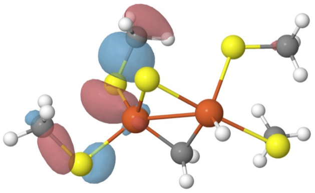

[HFe- 81] occ = 1.940

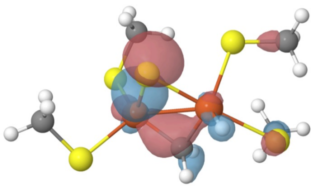

[HFe- 82] occ = 1.939

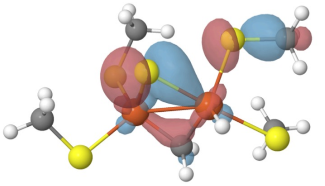

[HFe- 83] occ = 1.938

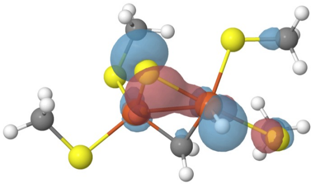

[HFe- 84] occ = 1.921

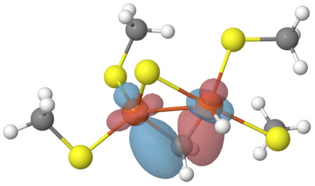

[HFe- 85] occ = 1.470

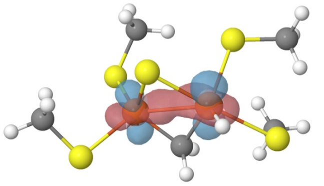

[HFe- 86] occ = 1.236

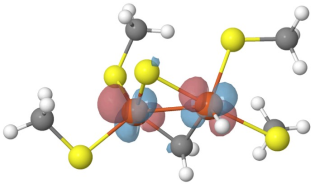

[HFe- 87] occ = 1.073

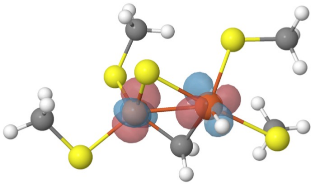

[HFe- 88] occ = 1.026

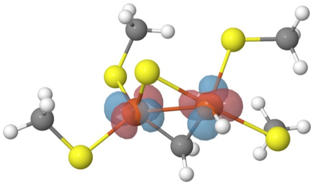

[HFe- 89] occ = 1.015

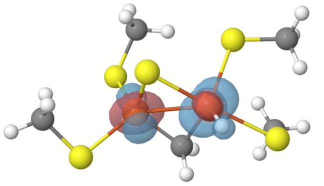

[HFe- 90] occ = 0.985

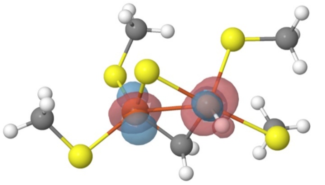

[HFe- 91] occ = 0.970

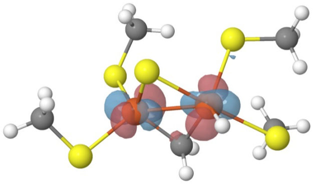

[HFe- 92] occ = 0.928

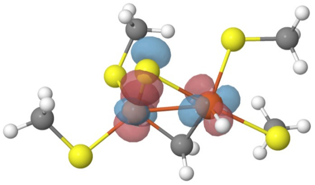

[HFe- 93] occ = 0.773

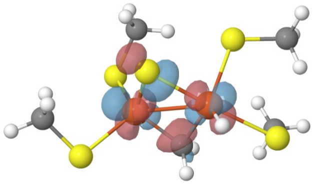

[HFe- 94] occ = 0.551

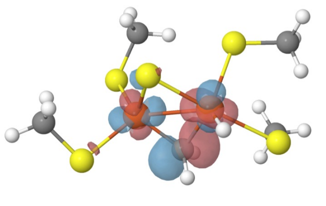

[HFe- 95] occ = 0.046

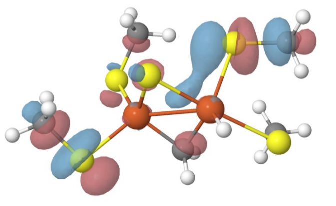

[HFe- 96] occ = 0.045

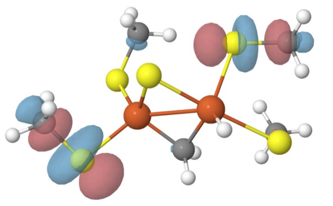

[HFe- 97] occ = 0.044

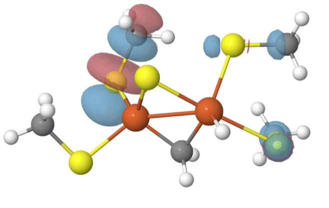

[HFe- 98] occ = 0.044

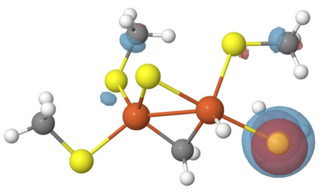

[HFe- 99] occ = 0.040

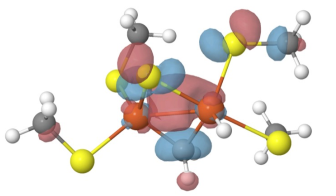

[HFe-100] occ = 0.032

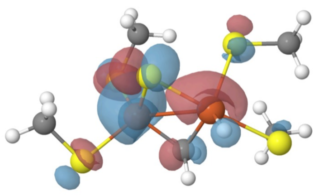

[HFe-101] occ = 0.031

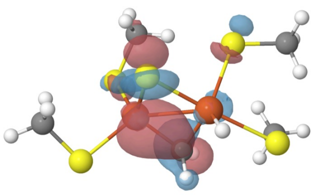

[HFe-102] occ = 0.030

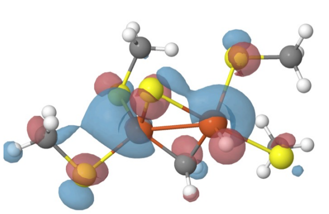

[HFe-103] occ = 0.028

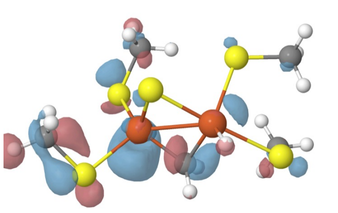

[HFe-104] occ = 0.028

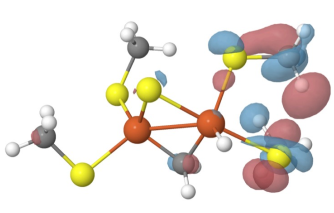

[HFe-105] occ = 0.027

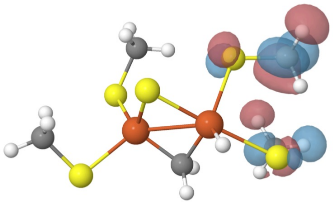

[HFe-106] occ = 0.026

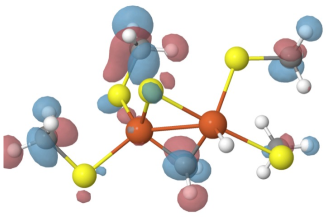

[HFe-107] occ = 0.026

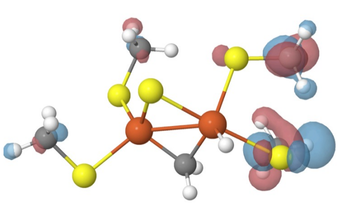

[HFe-108] occ = 0.026

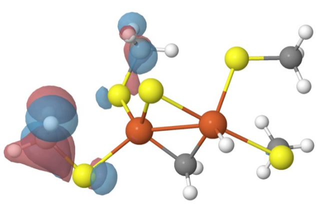

[HFe-109] occ = 0.026

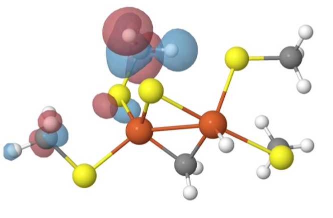

[HFe-110] occ = 0.025

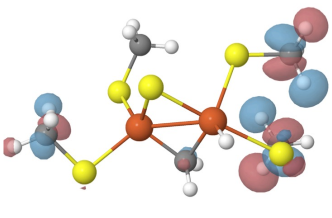

[HFe-111] occ = 0.025

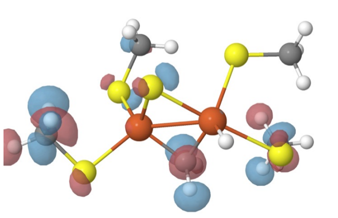

[HFe-112] occ = 0.025

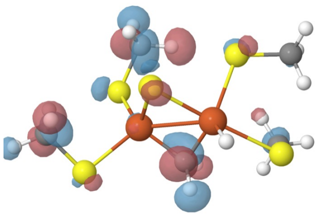

[HFe-113] occ = 0.024

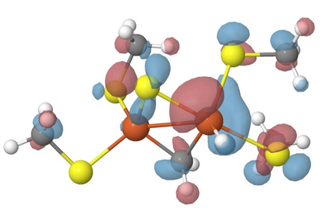

[HFe-114] occ = 0.023

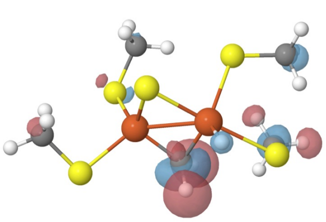

[HFe-115] occ = 0.023

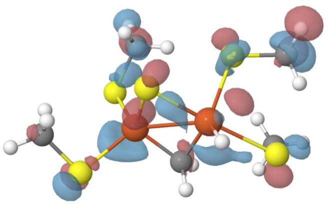

[HFe-116] occ = 0.023

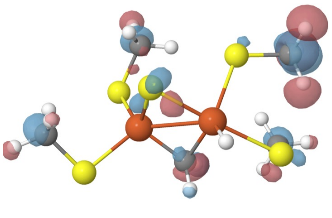

[HFe-117] occ = 0.022

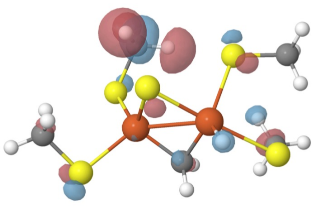

[HFe-118] occ = 0.022

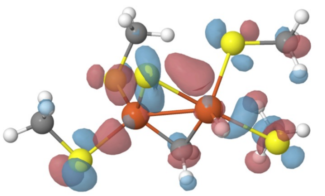

[HFe-119] occ = 0.022

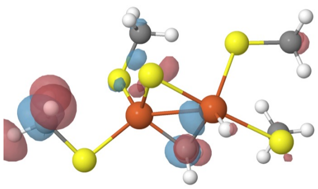

[HFe-120] occ = 0.021

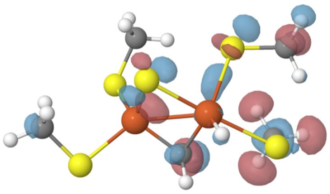

D. HFe_2

[HFe2- 58] occ = 1.960

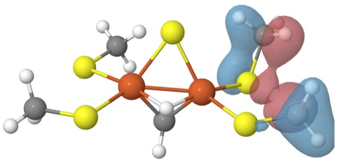

[HFe2- 59] occ = 1.960

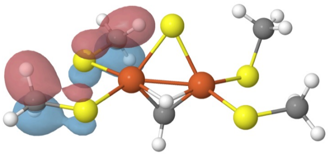

[HFe2- 60] occ = 1.959

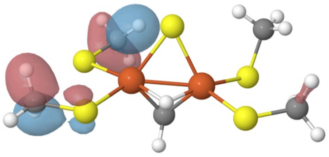

[HFe2- 61] occ = 1.959

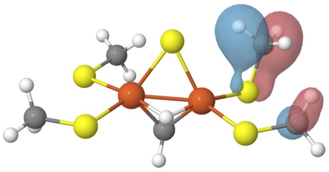

[HFe2- 62] occ = 1.959

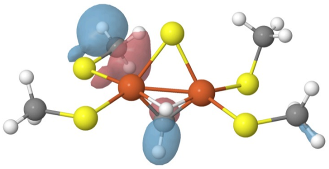

[HFe2- 63] occ = 1.959

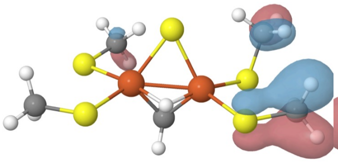

[HFe2- 64] occ = 1.958

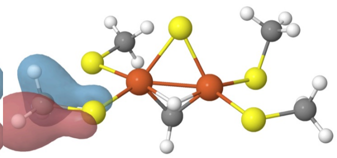

[HFe2- 65] occ = 1.957

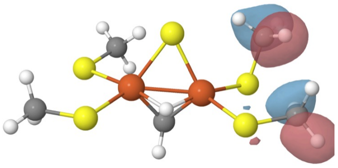

[HFe2- 66] occ = 1.956

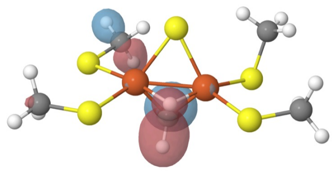

[HFe2- 67] occ = 1.954

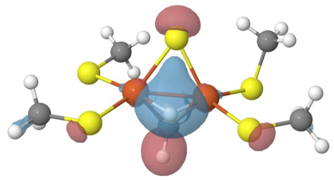

[HFe2- 68] occ = 1.951

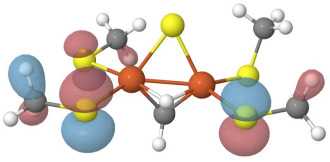

[HFe2- 69] occ = 1.951

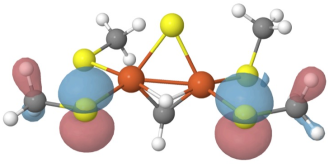

[HFe2- 70] occ = 1.951

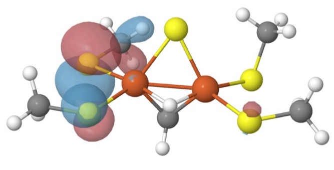

[HFe2- 71] occ = 1.950

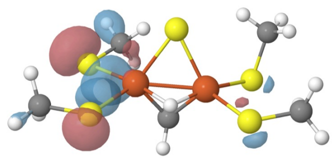

[HFe2- 72] occ = 1.949

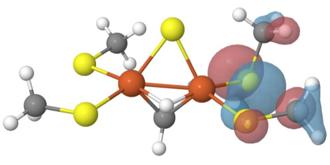

[HFe2- 73] occ = 1.949

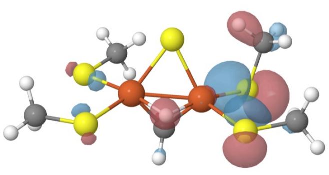

[HFe2- 74] occ = 1.948

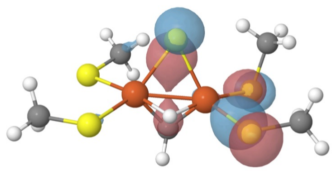

[HFe2- 75] occ = 1.947

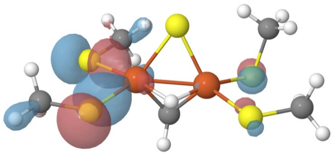

[HFe2- 76] occ = 1.946

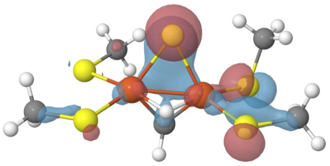

[HFe2- 77] occ = 1.944

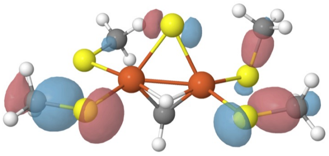

[HFe2- 78] occ = 1.943

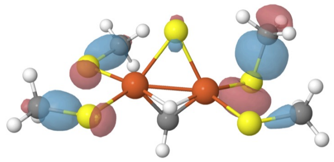

[HFe2- 79] occ = 1.943

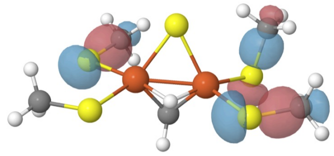

[HFe2- 80] occ = 1.942

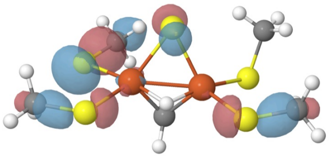

[HFe2- 81] occ = 1.941

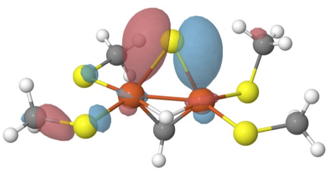

[HFe2- 82] occ = 1.939

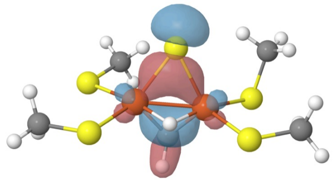

[HFe2- 83] occ = 1.933

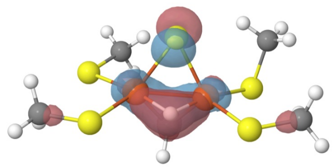

[HFe2- 84] occ = 1.919

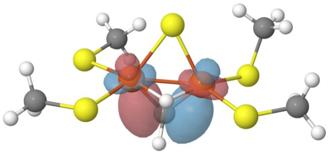

[HFe2- 85] occ = 1.532

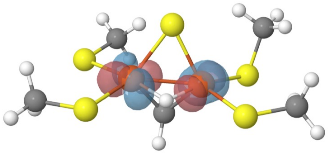

[HFe2- 86] occ = 1.486

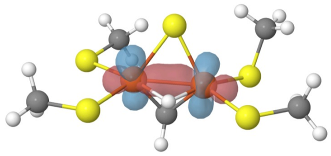

[HFe2- 87] occ = 1.212

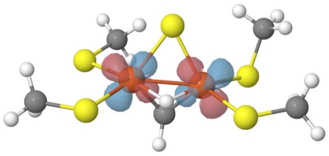

[HFe2- 88] occ = 1.030

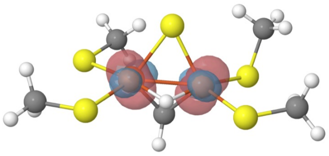

[HFe2- 89] occ = 1.019

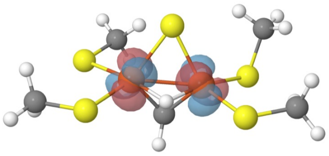

[HFe2- 90] occ = 0.978

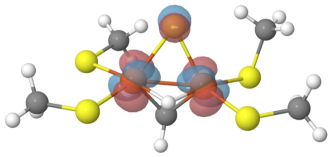

[HFe2- 91] occ = 0.962

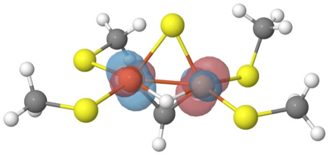

[HFe2- 92] occ = 0.793

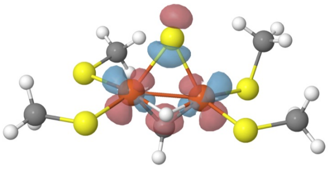

[HFe2- 93] occ = 0.534

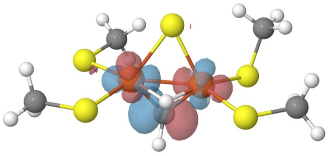

[HFe2- 94] occ = 0.487

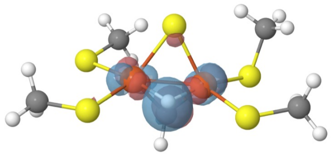

[HFe2- 95] occ = 0.047

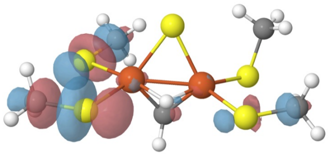

[HFe2- 96] occ = 0.045

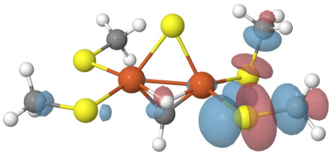

[HFe2- 97] occ = 0.044

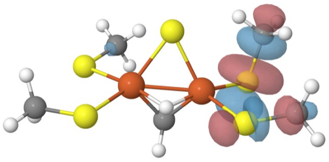

[HFe2- 98] occ = 0.044

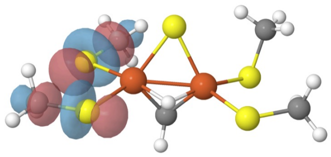

[HFe2- 99] occ = 0.036

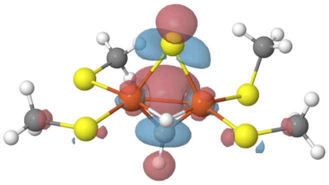

[HFe2-100] occ = 0.034

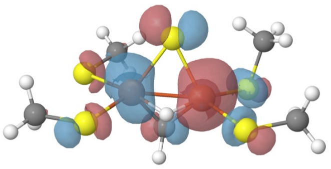

[HFe2-101] occ = 0.029

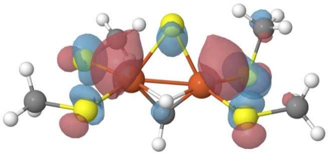

[HFe2-102] occ = 0.029

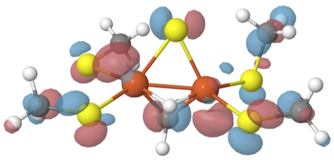

[HFe2-103] occ = 0.027

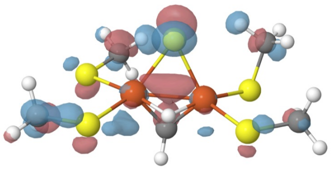

[HFe2-104] occ = 0.027

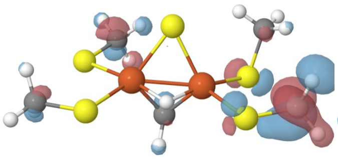

[HFe2-105] occ = 0.027

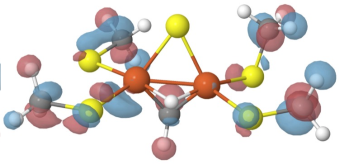

[HFe2-106] occ = 0.027

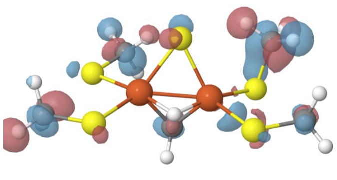

[HFe2-107] occ = 0.026

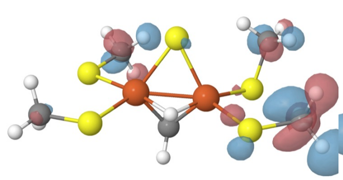

[HFe2-108] occ = 0.026

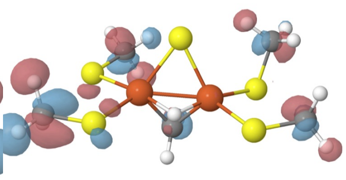

[HFe2-109] occ = 0.026

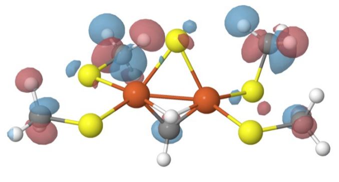

[HFe2-110] occ = 0.026

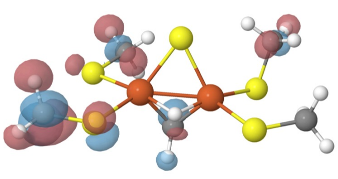

[HFe2-111] occ = 0.026

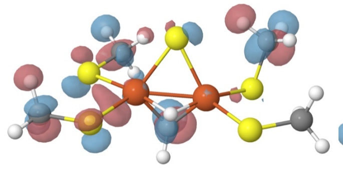

[HFe2-112] occ = 0.025

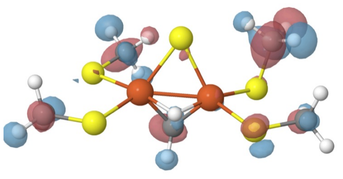

[HFe2-113] occ = 0.024

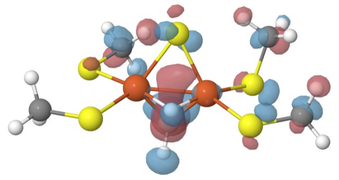

[HFe2-114] occ = 0.024

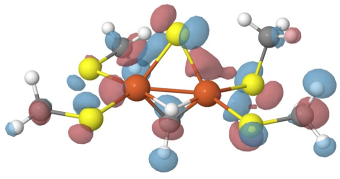

[HFe2-115] occ = 0.023

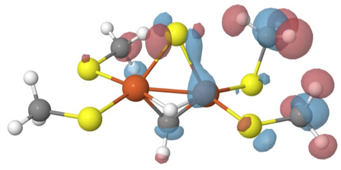

[HFe2-116] occ = 0.023

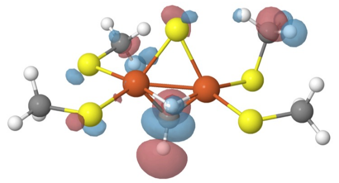

[HFe2-117] occ = 0.022

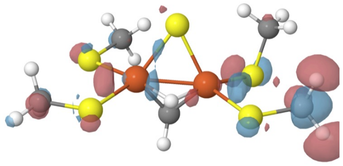

[HFe2-118] occ = 0.022

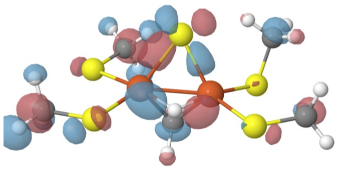

[HFe2-119] occ = 0.022

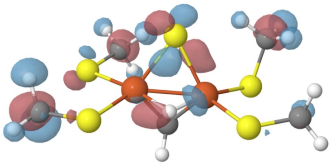

[HFe2-120] occ = 0.021

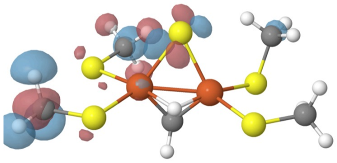

Supplement: Supplementary file 1 — jp3c06142_si_001.pdf [file jp3c06142_si_001.pdf]
